# Prevalence of experienced changes in artistic and everyday creativity in people with Parkinson’s disease

**DOI:** 10.1038/s41531-025-00924-1

**Published:** 2025-04-27

**Authors:** Blanca T. M. Spee, Julia S. Crone, Sirwan K. L. Darweesh, Marjan J. Meinders, Jozsef Arato, Young Ah Kim, Bastiaan R. Bloem, Matthew Pelowski

**Affiliations:** 1 Radboud University Medical Center, Donders Institute for Brain, Cognition and Behavior, Department of Neurology, Center of Expertise for Parkinson & Movement Disorders, Nijmegen, The Netherlands; 2 University of Vienna, Vienna Cognitive Science Hub, Vienna, Austria; 3 University of Vienna, Faculty of Psychology, Department of Cognition, Emotion, and Methods in Psychology, Vienna, Austria

**Keywords:** Psychology, Human behaviour, Quality of life

## Abstract

Creativity is the ability to generate novel and meaningful ideas or behaviors, encompassing both artistic originality and personal satisfaction. Emerging evidence suggests that people with Parkinson’s disease (PD) may experience changes in creativity. This study examines the prevalence of creativity changes in PD using cross-sectional data from the Netherlands (PRIME-NL, 2021–2023). Participants (*N* = 793) self-reported creativity changes, demographics, clinical factors, and pre-diagnosis creative engagement via a self-structured questionnaire. Descriptive analyses revealed that 41% of respondents reported creativity changes: 12% experienced an increase, 22% a decrease, and 7% fluctuations. Ordinal regression analysis showed that longer disease duration and dopamine agonists were associated with increased creativity, while older age and prior creative engagement predicted decreases. A sub-cohort (*n* = 292) reported creativity changes across seven domains, with changes most frequently observed in everyday creativity, sports/movement, and fine art/design. These findings underscore the need for further research on creativity in PD to inform person-centered treatment strategies.

## Introduction

Parkinson’s disease (PD) is characterized by a broad spectrum of motor, cognitive, and affective symptoms, which can greatly impact multiple facets of individuals’ lifestyles and activities^1,2^. Cognitive symptoms are often visible through difficulties in cognitive flexibility, divergent thinking skills and executive functioning^3–5^. Symptoms such as anxiety and apathy often reduce motivation for engaging in activities and participating in social interactions^6^. While these symptoms might be expected to reduce creative capacities^7–10^, some emerging evidence suggests a surprising opposite trend^11,12^. Case reports and clinical studies, particularly reported for the fine art domain, have indicated an increase in creative activities, including a higher propensity for engaging in creative pursuits^11,13–15^, self-reported boosts in creativity and in creative ability, and heightened motivation to participate in artistic practices^16^. Caregivers and healthcare professionals have also observed similar behavioral shifts, with some noting creative improvements^12^. These reports have been further supported by an initial survey study examining the prevalence of increased creativity among a Finnish population with PD^17^. Among the 280 respondents, initially contacted for research on impulse control disorder (survey opt-in rate of 75%), 19% reported an increase in artistic creativity after diagnosis. One-third of the respondents attributed these changes to dopamine agonist therapy.

This unexpected trend in PD offers a unique opportunity to examine our understanding of creativity in people with PD. Defining creativity is still a subject of considerable debate, discussed in various theoretical models, neuroanatomical frameworks, and assessed through diverse empirical methods^18–20^. In its simplest and most widely accepted definition, creativity encompasses the generation of novel and useful ideas or behaviors^18,21^, and is most commonly associated with expressive activity in art domains such as fine art, music or dance^21,22^. However, its scope extends far beyond traditionally recognized domains, including activities where original, innovative thinking, adaptability, or new combinations of knowledge are salient^7,19,22,23^. Recent perspectives argue that the traditional, externally focused definition overlooks the creator’s internal experience^24,25^. In many cases, the act of being creatively active does not necessarily produce a tangible product^26^; instead, it provides personal satisfaction and a sense of achievement (aesthetic learning process) that stands in stark contrast to merely repetitive behavior^27^. Researchers therefore suggest that ‘everyday creativity’ is often expressed in domestic activities such as cooking, gardening or handicraft, next to creative elements in sports and scholarly activities (science/technology)^24,25,28^.

The relationship between creativity or artistic changes and altered neurological conditions, along with clinical evidence of linking creative activity to dopaminergic treatment^2,16,29,30^, presents an intriguing but underexplored aspect of living with and treating PD^31,32^. However, current research lacks robust epidemiological evidence that not only assess the prevalence of creativity changes but also the direction—whether an increase, or a decrease, or fluctuations—across a large, representative sample. Existing surveys may also suffer from opt-in bias or fail to consider their actual focus on comorbidities, which could skew the reported prevalence of increased creativity. Additionally, there has been limited effort to integrate findings with demographics, clinical factors, and creative lifestyle prior to diagnosis.

The present study aims to systematically assess self-reported experience of creativity changes using the survey logistics and data from PRIME-NL, a longitudinal, nationwide prospective observation study in the Netherlands^33^. We explore (1) the prevalence of creativity changes in people with PD, whether they experience no change, an increase, a decrease, or fluctuations in creativity; (2) associations with demographics, clinical factors (disease duration, global cognition, disease severity, and dopaminergic treatment), and lifestyle factors to identify predictors of creativity change. From a sub-cohort, we report (3) potential domain-specific areas for creativity changes across seven creative domains^24,25^, which, in itself, marks a currently unexplored area of study on PD.

## Results

Demographics, clinical characteristics, and creative lifestyle before diagnosis, along with self-reported creativity changes as attributed by individuals to living with PD, are summarized in Table 1. The final sample included 793 participants. Opt-in rate for participation was 91% (total contacted = 916; 77 dropped due to increased disease severity/death or disinterest in continuing the PRIME-NL study; 42 excluded due to missing data on disease severity (Hoehn and Yahr (HY)-stage) and/or global cognition (MoCA^34^); four individuals did not respond to our creativity questions specifically). Creativity changes were reported along two medication classes, levodopa only/no medication and dopamine agonists as reported in Table 1 (for full description of brands and medication classes see Table S1 and for correlation heatmap Figure S1 in Supplementary Materials).

**Table 1:** Demographic, clinical characteristics, and creative lifestyle factors (*N* = 793) reported along experienced changes or no changes in creativity

Participant Factors	Total (% total or sub-group-wide mean)	Reported creativity change (% of total or sub-group-wide mean)^a^				
		no change	some change	Change type		
				increase	decrease	fluctuations
**Total**	*N* = 793 (100%)	470 (59.27%,) 95% CI [55.9, 62.7]	323 (40.72%)	94 (11.85%), 95% CI [9.6, 14.1]	171 (21.56%), 95% CI [18.8, 24.5]	58 (7.31%), 95% CI [5.5, 9.2]
**Gender**						
men	474 (59.77%)	300 (63.29%)	174 (36.71%)	45 (9.49%)	101 (21.31%)	28 (5.91%)
women	319 (40.23%)	170 (53.29%)	149 (46.71%)	49 (15.36%)	70 (21.94%)	30 (9.40%)
non-binary	0 (0%)	—	—	—	—	—
**Age (M)**	M = 70.96 ± 7.9	M = 71.02 ± 7.66	M = 70.88 ± 8.22	M = 68.70 ± 8.91	M = 72.07 ± 7.84	M = 71.02 ± 7.66
**Education**^**b**^						
Low	197 (24.84%)	130 (65.99%)	67 (34.01%)	12 (6.09%)	42 (21.32%)	13 (6.6%)
Medium	213 (26.86%)	118 (55.40%)	95 (44.60%)	31 (14.55%)	44 (20.66%)	20 (9.39%)
Higher	379 (47.79%)	219 (57.78%)	160 (42.22%)	51 (13.46%)	85 (22.43%)	24 (6.33%)
**Working situation**^**c**^						
Working	116 (14.63%)	76 (65.52%)	40 (34.48%)	14 (12.07%)	22 (18.97%)	4 (3.45%)
Not working	677 (85.37%)	394 (58.20%)	283 (41.80%)	80 (11.82%)	149 (22.01%)	54 (7.98%)
**Living situation**^**d**^						
Alone	112 (14.12%)	53 (47.32%)	59 (52.68%)	16 (14.29%)	30 (26.79%)	13 (11.61%)
With relatives	666 (83.98%)	409 (61.41%)	257 (38.59%)	75 (11.26%)	139 (20.87%)	43 (6.46%)
Facilitated care	15 (1.89%)	8 (53.33%)	7 (46.67%)	3 (20.00%)	2 (13.33%)	2 (13.33%)
**Clinical factors**						
Disease duration (years)	M = 7.08 ± 4.77	M = 6.91 ± 7.39	M = 7.75 ± 7.39	M = 8.64 ± 7.39	M = 6.43 ± 7.39	M = 7.88 ± 7.39
Global cognition (MoCA)	M = 18.17 ± 2.88	M = 18.12 ± 7.39	M = 18.22 ± 7.39	M = 18.89 ± 7.39	M = 18.05 ± 7.39	M = 17.71 ± 7.39
HY-stage (group *M*)^e^	M = 2.55 ± 1.14	M = 2.47 ± 7.39	M = 2.71 ± 7.39	M = 2.51 ± 7.39	M = 2.68 ± 7.39	M = 2.95 ± 7.39
HY- stage 1	158 (19.92%)	110 (69.62%)	48 (30.38%)	19 (12.03%)	24 (15.19%)	5 (3.16%)
HY- stage 2	268 (33.80%)	157 (58.58%)	111 (41.42%)	33 (12.31%)	60 (22.39%)	18 (6.72%)
HY- stage 3	166 (20.93%)	90 (54.23%)	76 (45.78%)	20 (12.05%)	41 (24.70%)	15 (9.04%)
HY- stage 4	171 (21.56%)	98 (57.31%)	73 (42.69%)	19 (11.11%)	39 (22.81%)	15 (8.77%)
HY- stage 5	30 (3.78%)	15 (50.00%)	15 (50.00%)	3 (10.00%)	7 (23.33%)	5 (16.67%)
**Medication**						
Levodopa	510 (64.31%)	316 (61.96%)	194 (38.04%)	40 (7.84%)	124 (24.31%)	30 (5.88%)
Dopamine agonist	244 (30.77%)	130 (53.28%)	114 (46.72%)	53 (21.72%)	37 (15.16%)	24 (9.84%)
No dopaminergic treatment	39 (4.92%)	24 (61.54%)	15 (38.46%)	1 (2.56%)	10 (25.64%)	4 (10.26%)
**Creative lifestyle prior to diagnosis**^f^	M = 3.84 ± 2.04	M = 3.49 ± 2.08	M = 4.37 ± 1.87	M = 4.24 ± 1.79	M = 4.37 ± 1.86	M = 4.59 ± 2.02

*Note:* Self-report survey conducted as focused survey within the prospective observational study PRIME-NL^33^. Data collection from 2021-2023. Ethnicity was not reported in detail as 99% were Dutch.

^a^For reporting the overall creativity change, participant answered based on reports to the question “Have you noticed changes in your own creativity or your desire to make something creative that you think are related to your life with Parkinson’s disease? These may include feeling less or more creative, as well as feeling the need to be creative (e.g., painting, drawing, writing, music, dancing, photography, creative gardening, sewing, etc.);” English translation provided by authors from original Dutch). To obtain confidence intervals for our categorical outcomes, we used the percentile bootstrap method^55^. We generated samples with replacement from our full dataset ( = 793), drawing 793 observations per resample, and calculated the obtained proportions for each outcome. We repeated this process 10,000 times, then we derived the confidence interval from the central 95% percentile of the bootstrapped proportion.

^b^Education was evaluated following Dutch school system standards into four categories, ordinal coding^33^: low education (in Dutch ‘geen’, ‘basisonderwijs’, ‘VMBO’, ‘MAVO’), medium education (in Dutch ‘HAVO’, ‘VWO’, ‘MBO’), higher education (in Dutch ‘HBO’, ‘Universiteit’, ‘PhD’), and level unknown (four individuals (0.50%) did not answer this question and were not included.

^c^Working status was categorized as ‘working’ (full-time employment, part-time employment, self-employed, education following not paid by employer, voluntary work) or ‘not-working’ (retired, unemployed, disabled, sickness benefit, active in household).

^d^Living situation used the three items: ‘alone’ (“I live alone.”), ‘with partner or family’ (“I live with my partner/my partner and children/family member other than partner”), or ‘in facilitated care’ (“I live in an institution/independently and receive outpatient supervision from a residential or welfare organisation (assisted living)/a house belonging to a residential or welfare organisation (sheltered living)”.

^e^HY-stage followed the standard scale and parameters for physical severity of the disease, see for details PRIME-NL study^1^.

^f^Creative lifestyle score based on 7-point Likert scale (“Before my diagnosis with PD, I participated in creative activities?” 1 = “not at all,” 7 = “very much”).

**Fig. 1:**
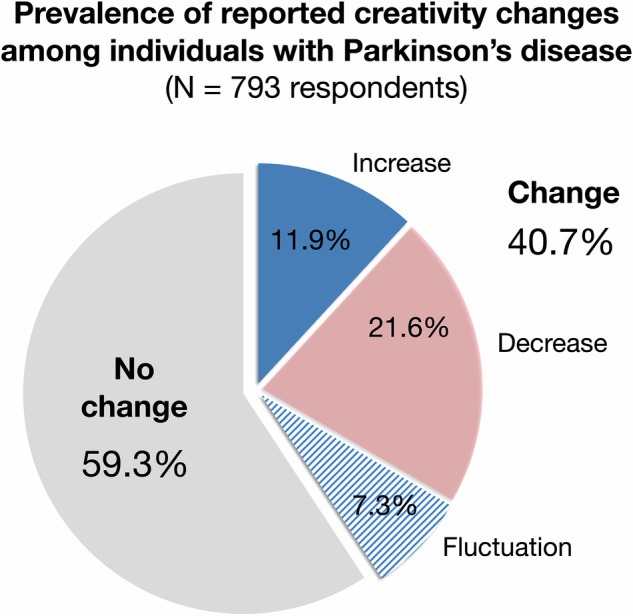
Prevalence and directionality of self-reported creativity change among individuals diagnosed with PD (*N* = 793). The chart shows the percentage of respondents who reported each type of change; gray – no change; blue – increase; red/rose – decrease; blue stripes – fluctuations. Due to rounding, the sum of individual percentages may not match the total exactly.

Among those who reported changes (*n* = 323), 29% (12% of full sample) reported increased, 53% (22% of full sample) indicated decrease; 18% (7% of full sample) reported fluctuating creativity. See Fig. 1 for prevalence of self-reported creativity changes among the PD-cohort.

The breakdown of participant characteristics (summarized in Table 1) suggested that women reported higher prevalence of changes as compared to men. Age showed no clear pattern regarding reporting creativity changes. However, participants reporting a decrease were on average three years older. Individuals having medium and high levels of education reported more changes than those with lower education levels. Individuals who were not working at the timepoint of the survey reported higher prevalence of changes as compared to working individuals. Individuals living alone reported more changes compared to those living with relatives or in care facilities. Notably, disease severity differed between individuals living alone, with relatives, or in care facilities with average HY-stages of *M* = 2.80 (*SD* = 1.06), *M* = 2.50 (1.15), and *M* = 3.69 (1.01), respectively. Participants who reported being more creatively active before their PD diagnosis were more likely to report changes in creativity compared to those who had a less active creative lifestyle.

Individuals reporting a change had on average a one year longer disease duration compared to those not reporting a change. Those reporting increase also had on average, about 1.5 years longer disease durations. Considering disease severity, participants with higher HY-stages (see Table S2 in Supplementary Materials for item structure to calculate HY-stages), particularly HY-3 and HY-5, reported the highest prevalence of creativity changes, while those with HY-1 showed the lowest prevalence (note, the number of participants was low within the HY-5 group). Although specific increases or decreases in creativity were not consistently associated with HY-stages, individuals with HY-1 had a lower prevalence of decreased creativity as compared to all other HY-stages.

Participants taking dopamine agonists showed a higher prevalence of creativity change as compared to those taking only levodopa or no dopaminergic medication. Individuals taking dopamine agonist also reported a higher prevalence of creativity increase and a lower prevalence of decrease, as opposed to levodopa only or to the no-medication group.

### Predictor Analysis of Creativity Change

To assess the association between reported changes and predictors, an exploratory series of stepwise generalized linear models was conducted with prevalence of change (categorical: no change/increase/decrease; ordinal regression coding) as the dependent variable. The final model (see Methods for full description; see D1 and Tables S3-S6 in Supplementary Materials for results of preliminary models), reported in Table 2, suggested that participants with a longer disease duration and those taking dopamine agonists were significantly more likely to report an increase in creativity. Older participants and those more engaged in creative activities before diagnosis were significantly less likely to report an increase and more likely to report a decrease. Participants’ gender, education, working or living situations, HY-score, global cognition (MoCA^34^) did not significantly predict changes. Notably, when comparing the significant predictors, dopamine agonist showed a weighted effect size (0.73) of roughly ten times that of other factors.

**Table 2:** Ordinal regression model predicting increased or decreased creativity changes among people with PD (*n* = 735)

			95% CI			
Fixed effects	Estimate	SE	Lower	Upper	*z*-value	Pr(>|z | )
Intercept 0/1^a^	−0.88	0.25	−1.36	−0.39	−3.56	**<0.001**
Intercept 1/2^a^	1.19	0.04	1.11	1.28	28.62	**<0.001**
Gender identity (women)^b^	0.16	0.17	−0.16	0.49	0.98	0.327
Age	−0.02	0.01	−0.05	−0.01	−2.22	**0.026**
Education^c^	0.11	0.09	−0.08	0.30	1.14	0.249
Working situation (working)^d^	−0.09	0.22	−0.52	0.35	−0.37	0.709
Living situation (with relatives)^e^	0.05	0.22	−0.38	0.49	0.25	0.805
Disease duration	0.04	0.02	0.01	0.08	2.40	**0.018**
MoCA^f^	−0.01	0.17	−0.35	0.32	−0.07	0.942
Disease severity^g^	−0.12	0.07	−0.27	0.02	−1.70	0.089
Creative lifestyle before PD diagnosis	−0.09	0.04	−0.16	−0.01	−2.29	**0.018**
Medication (dopamine agonist)^h^	0.73	0.19	0.36	1.10	3.90	**<0.001**

*Note:* Results based on final generalized linear model from an exploratory stepwise series using ordinal regression (statsmodels python library^52^); 58 individuals reporting creativity fluctuation excluded.

^a^Coding of creativity changes into three ordinal levels (0 = decrease, 1 = no-change, 2 = increase).

^b^Gender identity categorized into two groups (women = 1, men = 0).

^c^Education coded into three ordinal levels (low, medium, high; see Table 1 note for further documentation).

^d^Working situation coded into two categorical groups (working = 1, not working = 0).

^e^Living situation coded into two categorical groups (with partner/family = 1, alone/other = 0).

^f^MoCA, in line with former studies^53^, was divided into two categorical groups with a cut-off value of <= 17 representing cognitive impairments are present (encoded as 1); everything >=18 means no impairment (encoded as 0).

^g^HY-scores were coded into five ordinal levels reflecting disease severity.

^h^Medication coded into two categorical groups (levodopa only/no medication = 0, dopamine agonist = 1,). Note, that the levodopa-only and no-medication groups were combined for analysis due to the very low number of individuals in the latter category (*n* = 39) and based on literature suggesting that dopamine agonist use was the main hypothesized factor for creativity change^15^. For similar model excluding ‘no medication’ group, see Table S7 in Supplementary Materials. Ethnicity was excluded from the analysis due to very low numbers in one of the categories. See Methods for full description.

Distribution of creativity change across the significant independent variables are reported in Figure S2 for both medication types and Figure S3 for age, disease duration, and creative lifestyle before PD diagnosis in Supplementary Materials. None of the interactions between the found significant variables showed significance (see Table S8 in Supplementary Materials). Sensitivity analyses on medication confirmed the robustness of the final model, with medication remaining the strongest predictor across subgroups (see Description D1 and Tables S5-S6, S9-S10 in Supplementary Materials).

### Change along Creative Domains of Artistic or Everyday Creative Activities

**Fig. 2:**
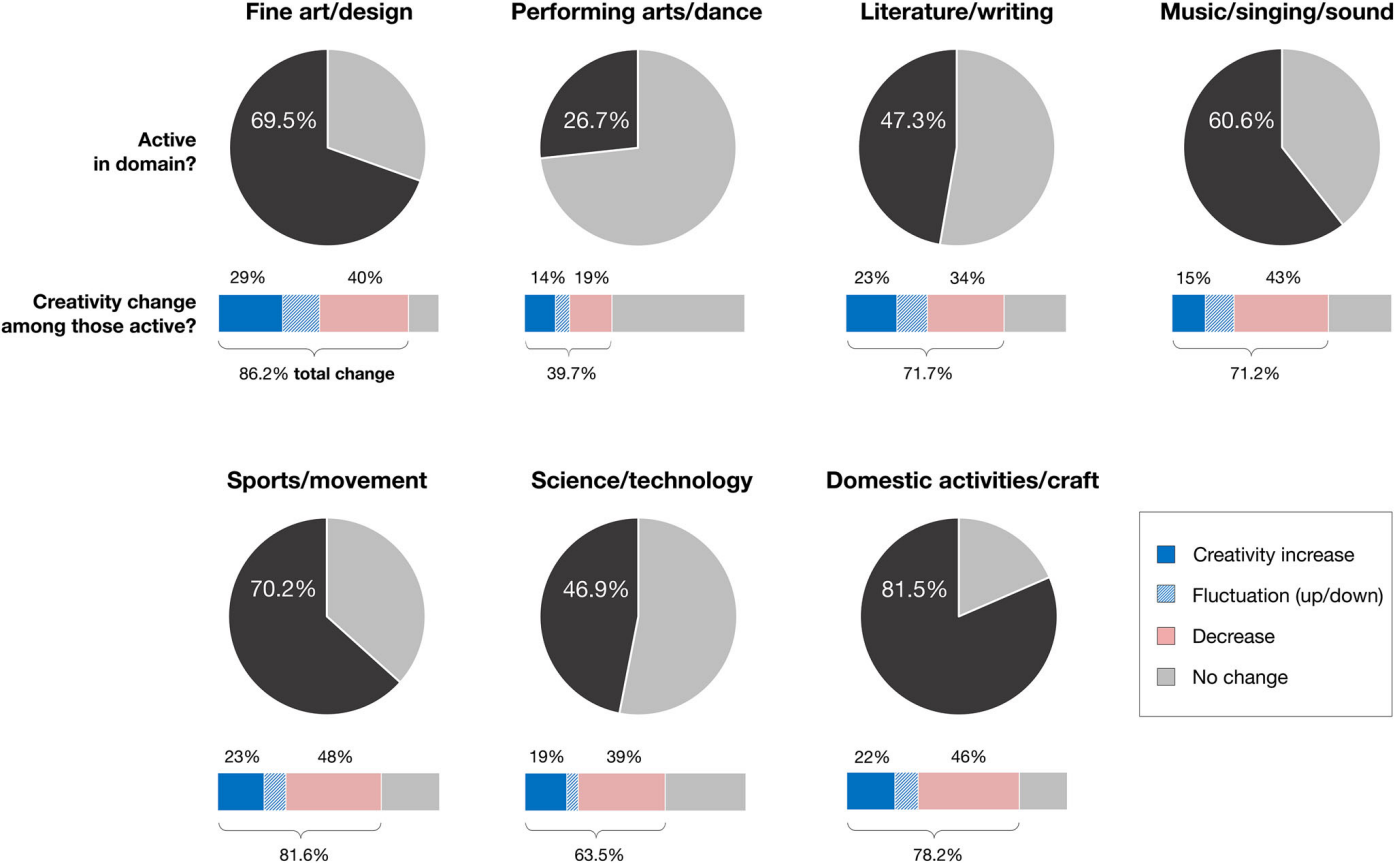
Domains of creative activities and prevalence of possible creativity change (*n* = 292). The chart shows the percentage of respondents who reported each type of change along a specific creative domain; gray – no change; blue – increase; red/rose – decrease; blue stripes – fluctuations. Individuals reporting a change independent of domain in prior main creativity question, opt-in 90%).

Seven domains for creative activities were assessed via a supplementary question following the main survey (see Methods)^25^. Following PRIME-NL requirements, this involved only participants who had reported some change and who were asked if they would like to answer additional items regarding this change. Individuals reported on both the specific domains in which they were active and the possible directionality of change for each selected domain. Results are summarized in Fig. 2. Descriptives statistics and demographic information of subgroup are reported in Table S11 in Supplementary Materials. The final sub-sample included *n* = 292 participants, constituting 90% of all individuals who had reported change (*n* = 31 opt-out, split generally across all change categories).

Of the seven possible domain categories, participants reported being active in, on average, *M* = 4.03 (*SD* = 1.93), and experienced some changes in *M* = 2.91 domains (*SD* = 1.54). Fourteen respondents (5%) reported no changes in any of the provided domains, and 14% (41 people) reported changes in only one domain. Participants were most active in everyday creative activities (sewing, crafts, interior and garden design, creative cooking, etc., >80%), followed by sport/movement, fine art/design, and music/singing/sound (all >60%). Literature/writing, science/technology, and performing arts/dance were comparatively lower.

The greatest creativity change was reported for fine art/design (86%), followed by sport/movement, everyday creative activities, literature/writing, and music/singing/sound (>70%). When looking to reported directionality (absolute increase divided by the sum of increased and decreased creativity, see Table S11 in Supplementary Materials), performing arts/dance and fine arts/design tended to show a higher proportion of change attributable to increased creativity, with, for example, 29% of the participants reporting increased creativity among those who had been active in fine arts/design. Similar patterns were found for literature/writing. Whereas science/technology, sports/movement, everyday creative activities, and music/singing/sound showed a comparatively lower proportion of change attributable to increased creativity versus comparatively higher proportion of decrease. Participants also tended to suggest the same directionality when reporting change across multiple domains, with conflicting (i.e., increase and decrease in two or more domains for the same individual) occurring in only 17% of cases.

## Discussion

We conducted a systematic epidemiological study on self-reported creativity changes in a large cohort of individuals living with PD. Of the 793 participants, 41% had experienced changes in creativity following their PD diagnosis. In this cohort (unbiased regarding art, creativity, and not part of another comorbidity study, see Methods), 12% reported increased creativity, 22% decreased creativity, and 7% noted fluctuations. We observed that according to descriptives, women, those with medium to higher education, individuals not currently working, as well as those with higher disease severity (HY-scores) reported a change more often. Subsequent predictor analyses identified that specifically longer disease duration and use of dopamine agonists (as opposed to levodopa only) were associated with increased creativity, while older individuals and those who were more creatively active before their PD diagnosis were more likely to report decreased creativity. Notably, intake of dopamine agonists showed an effect size roughly ten times higher than all other predictors.

Our findings expand on previous case reports (see for review^12^), clinical studies^2,10,30^, and a prior survey^17^ putatively linking PD to changes in creativity. Notably, the prevalence of creativity change is higher than previously reported^17^, perhaps suggesting under-reporting of total changes. Given that post-adolescence shifts in creativity or artistic interest^35–37^ are considered rare in the general population and for most neurodegenerative disorders^12^, these results offer important epidemiological insights for neuropsychological research and neuroscience in PD^4,9,10,12,38^.

The prevalence of creativity decrease is also of note. Although decrease in artistic motivation/output or in general felt visual creativity has been generally reported for PD, especially case evidence tended to emphasize only increase^13,15,39,40^. This may be tied to some general reporting bias or interest among practitioners and people with PD, tending to focus on the more compelling claims of artistic emergence or development^12^. This aspect of decrease or fluctuation, which to date had not been systematically assessed, presents a more complex picture and may also be a salient feature in many individuals dealing with PD.

Our results further support previous research linking changes in creative behavior to alterations in the dopaminergic system, influenced by both medication and neurodegeneration in PD^10,14,27,30,41^. The distinct impact of dopamine agonists, particularly those selective for D2/D3 receptors contrasts with the minimal impact of levodopa on creativity. Dopamine agonists could increase the variability of connectivity patterns in prefrontal regions and striatal areas of the brain’s reward system, though these effects likely depend on local baseline dopamine levels and may be tied to phasic variations in dopamine concentration. The finding of increased creativity with longer disease duration is more difficult to interpret. Higher dosages of levodopa or combinations of dopaminergic treatments might explain this increase. However, more empirical research is needed to clarify how different medications and dosages affect creativity.

The relation of factors to decreased creativity also aligns with previous studies. Two large epidemiological studies on PD and occupations have reported a lower prevalence of artistic jobs in late life prior to PD diagnosis^42,43^. While this may suggest that creative activities could be protective, it may also indicate a loss of interest in creativity during the prodromal phase, with potential recovery upon dopaminergic treatment. Creativity loss, followed by a rebound, may be more pronounced in individuals with a higher baseline of creativity. To further investigate this, we conducted an additional ordinal regression analysis examining the interaction between dopamine agonist use and prior creative engagement on self-reported creativity change (see Table S8 in Supplementary Materials). We found that while individuals who were creatively active before diagnosis were more likely to report a decrease in creativity and vice versa, the interaction term (DA agonist × prior creative engagement) was not significant. This suggests that in our study dopaminergic treatment is associated with increased creativity regardless of creative activity before PD diagnosis. Alternatively, those previously more creatively active may be more sensitive to subtle cognitive and motor declines that impact creative expression.

Our sub-sample findings showed a wide spread of reported creativity changes across various domains, with increases especially in visual arts/design, sports/movement, literature/writing, and everyday creative activities. This breadth of creative expressive avenues, the propensity for individuals to often note multiple domains, and the prominence of everyday creative activities, highlights the importance of considering a wider range of areas when investigating creativity and more broadly exploring the everyday lives and activities of people. The higher prevalence of creativity changes among women suggests that future research should pay closer attention to epidemiological aspects that may have been overlooked when considering lifestyle changes and treatment strategies. Personalized programs that encourage to ‘find your own creativity’ could cater a wider range of interests and abilities, which support to integrating such practices into daily lives of people with PD, offering practical and accessible means to increase well-being and quality of life^44,45^.

Our study has some limitations. Our sample lacked ethnic diversity, and we could not account for socioeconomic factors like income due to data security restrictions. A limitation is that we only had information on medication two years prior to the current survey. A sensitivity analysis using nationwide data showed that there were relatively modest changes in medication use patterns over a two-year period (please see Description D1 and for full statistics Table S9-S10 in Supplementary Materials). Although our participant pool was large and not biased by the study of co-morbidities, our study was conducted within the PRIME-NL study cohort. This cohort is divided into two groups for which one group has been starting to receive a different and new multidisciplinary healthcare access than the other group, which received usual care. During our study, the access to this new healthcare was only gradually implemented (see Methods). Nonetheless, differences in care as well as geographic and socio-cultural differences could have influenced findings, especially considering demographics. A sensitivity analysis on region of residence revealed that only medication remained significant in both regions (see Methods and for sensitivity analysis Description D1 and Tables S12-S13 in Supplementary Materials). In the usual care region, age and prior creatively active, also remained significant. Additionally, women and individuals with higher education were more likely to report an increase. However, we note that regional differences are not the focus of our study, and we suggest that these findings should be further investigated in future research. A further consideration in this study is the reliance on self-reported data to assess changes in creativity, which may be subject to recall bias and individual interpretation. However, self-reporting is a widely accepted method for assessing subjective experiences, particularly in clinical and epidemiological research^46–49^. While the absence of a validated multi-item scale may limit measurement precision, our study aimed to estimate the prevalence of self-reported creativity changes in a large PD cohort rather than quantify creativity itself. Future research could benefit from standardized, multi-item assessments to provide a more detailed evaluation of creative engagement over time. Additionally, longitudinal studies may provide further insight into the dynamics of creativity changes in PD, examining the influence of disease progression from the prodromal phase through the various stages of clinically manifest PD and of dopaminergic treatment. Such studies could provide valuable insights into the use of creativity changes as personalized lifestyle diagnostic markers of disease progression (creativity decrease) and medication efficacy (creativity increase), potentially improving personalized PD care and treatment strategies.

Overall, our study advocates for a person-centered view to PD management, where creativity is not just an ancillary concern but a core aspect of healthcare that deserves further attention and research.

## Methods

We conducted an exploratory, epidemiological study using self-structured questions within the PRIME Parkinson Evaluation Study^33^ (PRIME-NL), a nationwide prospective observation study in the Netherlands. PRIME-NL assesses the impact of an innovative healthcare model, PRIME Parkinson^50^, which has been gradually implemented in Central Gelderland since 2021, with full roll-out planned 2025 (baseline assessed 2020). The study compares outcomes in this region to those in areas providing standard care using comprehensive healthcare data and annual surveys (see PRIME-NL protocol for full methodology^33^).

### Participants

The study included all participants taking part in the PRIME-NL Study from March 2021 to March 2023. Inclusion criteria included a clinical diagnosis of parkinsonism, excluding drug-induced parkinsonism, and those who had visited a Neurology outpatient clinic of a PRIME-NL affiliated community hospital center at least once during 2020. Participants needed to be willing and able to provide written informed consent.

### Procedure

All participants had provided PRIME-NL pre-obtained written informed consent. As part of the annual PRIME-NL survey, researchers could submit short lists of targeted questions (focused survey), including ours, which were selected by the PRIME-NL advisor board. Our study involved two rounds of assessment (conducted annually 2022 to 2023), with data combined for the same individuals for the present analysis. Participants in the PRIME-NL program were contacted via post mail, email, or SMS and asked to complete the full questionnaire, which could be done either online, paper-pencil (at home), or via tele-support provided by a researcher. The entire annual PRIME-NL survey took about two hours, with our questions taking approximately five minutes.

The study protocol was approved by the Radboud University Medical Center Ethics Board (CMO file numbers 2019-5618 for PRIME-NL and 2021-12985 for present study) and was performed in accordance with the ethical standards laid down in the 1964 Declaration of Helsinki and its later amendments.

### Survey Questions

#### Prevalence of creativity change

To estimate the prevalence of self-reported changes in creativity related to PD, we first asked participants: “Have you noticed any changes in your own creativity or your desire to create something creative, which you think are related to living with Parkinson’s disease? This could be feeling less or more creative, but also to engage and the need to engage in creative activities (such as painting, drawing, writing, music, dancing, photography, creative gardening, sewing, etc.).” This phrasing was selected to provide a general measure of creative feeling/expression across domains. Participants could answer by selecting one of four options: no change, increase, decrease, and fluctuations, latter means experiencing increases and decreases.

#### Demographic factors

As demographic factors we included gender identity, age, ethnicity, education, working situation, and living situation (see specific categorizations in Table 1 Note). Additionally, we recorded the healthcare region information (usual care versus PRIME care region). These data were retrieved from the 2023 PRIME-NL data.

#### Clinical/medication factors

Factore included disease duration from time of initial diagnosis (in years), disease severity (measured via the HY-stages. The HY-stages were based on six questions from the Movement Disorder Society Unified Parkinson’s Disease Rating Scale (MDS-UPDRS^51^) and two self-structured items (see Table S2 in Supplementary Materials, for HY-stages categorization parameters). Global cognition was assessed using the Montreal Cognitive Assessment, MoCA^34^. All factors were retrieved from 2023 PRIME-NL data.

Medication/dopaminergic treatment records were obtained by the treating neurologist at the beginning of the PRIME-NL project in 2020. Medication classes for analysis were divided into two groups: intake of (1) dopamine agonist independent of additional levodopa intake and intake of (2) levodopa only/no medication (other drug information was obtained but was not part of our analysis, see Table S1 in Supplementary Materials). Note, that the levodopa-only and no-medication groups were combined for analysis due to the very low number of individuals in the latter category (*n* = 39) and based on literature suggesting that dopamine agonist use was the main hypothesized factor for creativity change^15^.

#### Creative lifestyle before PD diagnosis

This was assessed via one self-reported questions using a 7-point Likert scale (*“Before my diagnosis with PD, I participated in creative activities?”* 1 = *“not at all”* to 7 = *“very much”*). This information was obtained in the earlier round of the survey, 2021-2022.

#### Creative domains

Finally, specific domains for experienced creativity changes were assessed via the Inventory of Creative Activities and Achievements^20,24^, which included a list of artistic and everyday life modalities where individuals might pursue creative outputs or activities, including the following domains: (a) fine art or design (drawing, creative photography, sculpture, woodwork, etc.), (b) performing arts (acting, devising choreography, dance, drama, etc.), (c) literature and writing (texts, writing, blogs, poems, etc.), (d) music, singing, and sound (composing or adapting melodies, making sounds, etc.), (e) sports and exercise (learning or inventing new tricks or moves, sports training, etc.), (f) science/technology (solving technical problems, computer programming, etc.), and (g) everyday creative activities (sewing, crafts, making your own cards, rugs, bags, interior and garden design, cooking, social (devising games, organizing parties, etc.). Descriptions, as given above, were the exact wordings presented to participants (although some separate items from the original survey were combined into one ‘everyday creative activities’ category for time of questioning reasons).

The creative domains were included as follow-up items (as additional to the ones asked within the PRIME-NL survey) for those who had reported some creativity change in the main survey in the 2022–2023 rounds, and thus was not completed by all participants (see Table S11 in Supplementary Materials for sub-sample demographics).

### Statistical Analysis

#### General prevalence

Descriptive statistics (prevalence in absolute numbers and percentage) were used to quantify the overall prevalence, as well as the directionality (no change, increase, decrease, fluctuations), of reported creativity changes among participants, as well as along all factors.

#### Assessment of predictors of changes

The analysis of predictors of creativity change was conducted using an exploratory stepwise series of generalized linear models (see Description D1 in Supplementary Materials), as follows: Initially, we performed a logistic regression to model creativity change as a binary outcome (yes—including data from increase, decrease, and fluctuations—versus no change). This model (see Table S3 in Supplementary Materials) included predictors gender identity, age, education, working situation, living situation, disease duration, MoCA scores, HY-stages, and prior creative lifestyle. As a second step we conducted an ordinal regression (using the statsmodels python library^52^, Table S4 in Supplementary Materials), which was used for the version including directionality data coding the creativity changes into three categories (0 = decrease, 1 = no-change, 2 = increase) with the same set of predictors. We excluded the fluctuations category in our ordinal regression analysis of creativity changes to enhance clarity and interpretability. Excluding fluctuations also addressed potential concerns about measurement reliability and operational definition, ensuring the robustness of our findings. Hence, we only reported fluctuations within the descriptive statistics reports.

Our final model (see Table 2) was an ordinal regression version including directionality data from our latest measurement timepoint coding the creativity changes into three categories (0 = decrease, 1 = no-change, 2 = increase) with the predictors: gender identity, age, education, working situation, living situation, disease duration, MoCA scores, HY-stages, creative lifestyle before PD diagnosis, and medication. All predictors were mean-centered before fitting the ordinal and logistic regression models.

Independent variables were included into the models as follows: gender identity categorized into two categorical groups (women = 1, men = 0); education coded into three ordinal levels (low, medium, high; see Table 1 note for further documentation); working situation coded into two categorical groups (working = 1, not working = 0); living situation coded into two categorical groups (with partner/family = 1, alone/other = 0); disease duration in years; due to the skewed distribution of the MoCA results, we categorized the MoCA, in line with former studies^53^, into two categorical groups with a cut-off value of <= 17 representing cognitive impairments are present (encoded as 1); everything >=18 means no impairment (encoded as 0). HY-scores were coded into five ordinal levels reflecting disease severity (1 = unilateral involvement, 2 = bilateral, without balance issues, 3 = mild to moderate bilateral, postural instability, physically independent, 4 = severe disability, still able to walk or stand unassisted, and 5 = wheelchair bound or bedridden unless aided; see Table S2 in Supplementary Materials); creatively active before PD-diagnosis was included as ordinal variable using a 7-point Likert scale; medication coded into two categorical groups (dopamine agonist = 1, levodopa only/no medication = 0).

#### Sensitivity analyses

As an intermediary step in the above assessment, we conducted a sensitivity analysis to examine the effects of medication and of recruitment region on change outcomes (see Supplementary Materials D1 for full explanation). The analysis on the influence of medication was conducted to explore potential impact on the regression model’s other coefficients given the known impact of dopamine agonists on other cognitive processes. As the data on medication intake was up to two years old at the time of the collection of other data, we also wished to consider results with and without medication. As reported in Tables S5-S6 in Supplementary Materials, in the dopamine agonist group, no predictors reached traditional levels of statistical significance, and the coefficient for disease duration reduced to 0.04 from 0.06. In the levodopa-only group, only prior creative activity remained significant. These findings supported including medication as a variable in the final model reported in Table 2.

Additionally, we want to ensure that medication changes within a two-year period in a similar cohort is low to modest. We retrieved data from a national claims database held by Vektis, which contains the diagnostic and treatment data of more than 99% of the population of the Netherlands. We previously leveraged this database to study the association between levodopa initiation and mortality in Parkinson’s disease^54^. Results are reported in Description D1 and full statistics in Tables S9–S10 in Supplementary Materials). Taken together, these are relatively modest changes in medication use over a two-year period. We therefore included the medication data in our final model (see Table 2).

We conducted a second sensitivity analysis comparing healthcare region differences (i.e., PRIME versus usual care region, see Tables S12-S13 in Supplementary Materials).

## Supplementary information

Supplementary Materials

## Data Availability

The datasets generated and/or analyzed during the current study are available in the github repository, https://github.com/blancaspee/prevalence_creativity_parkinson.git.
