# Supplementary material for: Prevalence of experienced changes in artistic and everyday creativity in people with Parkinson’s disease
Source: NPJ Parkinsons Dis. 2025 Apr 27;11:97. doi: 10.1038/s41531-025-00924-1 (PMC12034792; doi:10.1038/s41531-025-00924-1)

Supplementary Materials

Prevalence of Experienced Changes in Artistic and Everyday Creativity in People with Parkinson's Disease

Blanca T.M. Spee[1,2,3,*], Julia S. Crone[2], Sirwan K.L. Darweesh[1], Marjan J. Meinders[1], Jozsef Arato[2], Young Ah Kim[3], Bastiaan R. Bloem[1], Matthew Pelowski[2,3]

[1] Radboud University Medical Center, Donders Institute for Brain, Cognition and Behavior, Department of Neurology, Center of Expertise for Parkinson & Movement Disorders. Nijmegen, The Netherlands.

[2] University of Vienna, Vienna Cognitive Science Hub, Vienna, Austria.

[3] University of Vienna, Faculty of Psychology, Department of Cognition, Emotion, and Methods in Psychology, Vienna, Austria.

* Corresponding Author: blanca.spee@radboudumc.nl, blanca.spee@univie.ac.at

Supplementary Descriptions of Data Analyses and Sensitivity Analysis

D1. Steps to Final Ordinal Regression Model

1. Logistic Regression Analysis for Yes/No Self-Reported Creativity Change

In the logistic regression analysis, a final sample of 735 participants was included (M_{age} = 70.93, *SD* = 7.89; age-range = 49; 60.10 % men, 39.90 % women; $M_{HY\text{-}score}$ = 2.54 +/- 7.39; M_{MoCA}= 18.20+/-2.86). For the correlation heatmap, see Fig. S1 in the Supplementary Materials. Our logistic regression analysis examined (see Table S3, Supplementary Materials) revealed that the Hoehn and Yahr (HY)-stages, indicating physiological disease severity, showed a positive trend (B = 0.13, SE = 0.17, z = 1.84, p = .066), suggesting higher HY scores are linked to an increased likelihood of reporting a change in creativity. Living situation emerged as a significant factor (B = -0.41, SE = 0.21, z = -1.97, p = .049), with its negative estimate indicating that living alone is associated with a decreased likelihood of reporting a change in creativity. Most notably, prior creative activity was a highly significant predictor (B = 0.21, SE = 0.04, z = 5.43, p < .0001), demonstrating a strong association between previous creative engagement and the likelihood of experiencing a change in creativity. Other variables, including gender identity, age, disease duration, education, working, and living situation, and MoCA scores, did not show significant associations; however, higher education showed a trend towards reporting a creativity change (B = 0.18, SE = 0.10, z = 1.86, p = .062).

2. Ordinal Regression Analysis for Directionality

To investigate directionality, an ordinal regression analysis was conducted, coding creativity change along three categories: 0 = decrease, 1 = no-change, 2 = increase (for a full report, see Table S4 in Supplementary Materials). Additionally, the 'fluctuations' category

was excluded leading to a final sample of 735 participants for this analysis. The independent variables were the same as those in the logistic regression.

Among the predictors, age showed a significant negative association (B = -0.03, SE = 0.01, z = -2.68, p = .007), indicating that older participants were less likely to report an increase in creativity. Disease duration was positively associated with an increased likelihood of reporting an increase in creativity (B = 0.06, SE = 0.02, z = 3.82, p < .001). Again, prior creative activity was a significant predictor (B = -0.08, SE = 0.04, z = -2.20, p = .028), however with a negative association suggesting that those more creatively active in the past were less likely to report a decrease in creativity at the time of assessment. Other variables, including gender identity, MoCA, HY-score, education, working situation, and living situation, did not reach statistical significance.

3. Sensitivity Analyses

Two sensitivity analyses were conducted on the ordinal regression model, including directionality (see Table S5-S6 and Table S12-S13 in Supplementary Materials). The first sensitivity analysis looked at medication intake, exploring if significant predictors in the model would lose significance or show a reduced coefficient when considering dopamine agonists and levodopa classes. The second sensitivity analysis compared the PRIME-NL region with the usual care region to check if healthcare region differences impacted the results.

Medication: In the dopamine agonist group (see Table S5-S6, Supplementary Materials), no predictors reached traditional levels of statistical significance, and the coefficient for disease duration reduced to 0.04 from 0.06. In the levodopa-only group (see Table S5 in Supplementary Materials), only prior creative activity remained significant (B = -

0.10, *SE* = 0.04, *z* = -2.19, *p* =.0.03). These findings led to including medication as a variable in the final model reported in Table 2 (main manuscript).

Additionally, we want to ensure that medication changes within a two-year period in a similar cohort is low to modest. We retrieved data from a national claims database held by Vektis, which contains the diagnostic and treatment data of more than 99% of the population of the Netherlands. We previously leveraged this database to study the association between levodopa initiation and mortality in PD[4]. As for (i) 6.4% of individuals who were using levodopa and/or a dopamine agonist in 2019 were no longer using either of these medications in 2021. As for (ii) 2.5% of those who were on no medication in 2019 started taking levodopa and/or a dopamine agonist by 2021. As for (iii) 5.8% of individuals who were taking only levodopa in 2019 switched to a dopamine agonist or a combination of dopamine agonist and levodopa in 2021, whereas (iv) 4.4% (*n* = 1,077) of those who were taking both levodopa and a dopamine agonist in 2019 switched to only levodopa in 2021. (v) 2.5% (*n* = 605) of those who were on no medication in 2019 started taking levodopa and/or a dopamine agonist by 2021 (please see full statistics Table Table S9-S10 in Supplementary Materials). Taken together, these are relatively modest changes in medication use over a two-year period. We therefore included the medication data in our final model (see Table 2).

Region of Residence: In the PRIME-NL region (see Table S12, Supplementary Materials), only medication remained significant (*B* = 1.06, *SE* = 0.40, *z* = 2.65, *p* =.008). In the usual care region (see Table S13, Supplementary Materials) age, prior creative activity, and mediation remained significant. Additionally, gender identity became significant, indicating that women are more likely to report an increase in creativity (*B* = 0.45, *SE* = 0.21, *z* = 2.07, *p* = .038) and education (*B* = 0.32, *SE* = 0.13, *z* = 2.43, *p* = .015) indicating that higher education led to a higher likelihood of reporting an increase.

Table S1. Brands of drugs and medication classes

Category number	Brand	Medication class
	**levodopa**	
0	Levodopa/Carbidopa (without gastric acid resistance)	levodopa
1	Levodopa/Carbidopa (with gastric acid resistance)	levodopa
2	Liquid intestinal levodopa/Carbidopa	levodopa
3	Levodopa/Carbidopa/Entacapon	levodopa
4	Levodopa/Carbidopa/Tolcapon	levodopa
5	Levodopa/Bensarazide (without gastric acid resistance)	levodopa
6	Levodopa/Bensarazide (with gastric acid resistance)	levodopa
	**dopamine (DA)-agonist**	
7	Pergolide	DA-agonist
8	Pramipexol	DA-agonist
9	Ropinirol	DA-agonist
10	Apomorfine	DA-agonist
11	Rotigotine	DA-agonist
12	Bromocriptine	DA-agonist
13	Piribedil	DA-agonist
	**other**	
14	Entacapon	other: COMT inhibitor
15	Toolcapon	other: COMT inhibitor
16	Biperideen	other: anticholinergics
17	Trihexyfenidyl	other: anticholinergics
18	Rasagiline	other: MAO inhibitor
19	Selegiline (tablets)	other: MAO inhibitor
20	Safinamide	other: MAO-B inhibitor
21	Amantadine	other: NMDA antagonist
22	Selegilne (sublingual)	other: anti-depressant
23	Mucana pruriens	other: food-supplement
24	Carbidopa	other: DOPA decarboxylase inhibitor
25	Propanolol	other: betablocker
26	Rivastigmine patch (mg/day)	other: antidementivum

Note: Medication/dopaminergic treatment records were obtained by the treating neurologist at the beginning of the PRIME-NL project in 2020. Due to lack of supporting literature, we did not separate enteral levodopa from levodopa administered intermittently for analyses. 39 participants did not take either main medication class, all participants who took medication, took either levodopa only (categories 0-6) or dopamine agonists (category 7.13). In the latter dopamine agonist group, most participants also took levodopa (only 18 were reported as taking dopamine agonists only).

Table S2. Item structure for calculating Hoehn & Yahr (HY) score.

HY-score	Description	Questionnaire	Item	Criteria
**HY 5**	wheelchair bound or bedridden unless aided	P9: UPDRS part II	walking and balance	*"I usually need support from someone to walk safely without falling."*
**HY 4**	severe disability; still able to walk or stand unassisted	P9: UPDRS part II	getting out of bed, out of a car, or standing up from a deep chair	*"I usually or always need help."*
		P9: UPDRS part II	getting out of bed, out of a car, or standing up from a deep chair	*"Getting out of bed, out of a car, or standing up from a deep chair."*
		P9: UPDRS part II	walking and balance	*"I usually use an aid (cane, walker) to walk safely without falling. However, I usually do not need someone to support me."*
**HY 3**	mild to moderate bilateral disease; some postural instability; physically independent	P9: UPDRS part II	getting out of bed, out of a car, or standing up from a deep chair	*"I need more than one attempt to get up, or I sometimes need help with it."*
		P9: UPDRS part II	walking and balance	*"I sometimes use an aid to walk, but I do not need any help from someone else."*
**HY 2**	bilateral involvement without impairment of balance	P9: questionnaire motor symptoms	bradykinesia left/right	left <10 & right <10
**HY 1**	unilateral involvement only	P9: questionnaire motor symptoms	bradykinesia left/-right	*"Having completed the three relevant questionnaires but not fulfilling any of the abovementioned criteria."*

Note. The HY-score was based on six questions from the Movement Disorder Society Unified Parkinson's Disease Rating Scale (MDS-UPDRS) and two self-structured items

Table S3. Logistic regression for general change in creativity (*N* = 793).

			95% CI			
Fixed effects	Estimate	SE	Lower	Upper	*z*-value	Pr(>|z|)
Intercept	-0.127	0.225	-0.568	0.313	-0.566	.572
gender identity (women)	0.222	0.161	-0.094	0.538	1.379	.168
age	-0.011	0.010	-0.032	0.009	-1.080	.280
education	0.179	0.096	-0.009	0.366	1.864	. 062
working situation (working)	-0.275	0.227	-0.721	0.171	-1.209	.227
living situation (with relatives)	-0.407	0.206	-0.811	-0.003	-1.972	**.049**
disease duration	-0.002	0.016	-0.034	0.030	-0.134	.894
MoCA	0.103	0.167	-0.225	0.430	0.613	.540
HY-score	0.133	0.073	-0.009	0.276	1.839	.066
creatively active before PD diagnosis	0.208	0.038	0.133	0.283	5.426	**.000**

Note. Results based on generalized linear model from an exploratory stepwise series (statsmodels python library[27]). Dependent variable: overall creativity changes (yes/no). Independent variables included gender identity, age, education, working situation, living situation, disease duration in years, MoCA, HY-score, and prior creatively active. Gender identity categorized into two groups (women = 1, men = 0); education coded into three ordinal levels (low, medium, high; see Table 1 note for further documentation); working situation coded into two categorical groups (working = 1, not working = 0); living situation coded into two categorical groups (with partner/family = 1, alone/other = 0); MoCA, in line with former studies[35], was divided into two categorical groups with a cut-off value of <= 17 representing cognitive impairments are present (encoded as 1); everything >=18 means no impairment (encoded as 0); HY-scores were coded into five ordinal levels reflecting disease severity. Ethnicity was excluded from the analysis due to very low numbers in one of the categories. See Methods for full description.

Table S4. Ordinal regression reporting creativity change along three categories (0 = decrease, 1 = no-change, 2 = increase) as dependent variable (*n* = 735).

			95% CI			
Fixed effects	Estimate	SE	Lower	Upper	*z*-value	Pr(>|z|)
Intercept 0/1	-1.169	0.233	-1.626	-0.713	-5.019	**.000**
Intercept 1/2	1.229	0.039	1.152	1.305	31.473	**.000**
gender identity (women)	0.191	0.163	-0.128	0.509	1.174	.240
age	-0.029	0.011	-0.050	-0.008	-2.679	**.007**
education	0.108	0.094	-0.077	0.292	1.145	.252
working situation (working)	-0.075	0.222	-0.510	0.360	-0.336	.737
living situation (with relatives)	0.084	0.212	-0.332	0.500	0.395	.693
disease duration	0.063	0.016	0.031	0.095	3.818	**.000**
MoCA	-0.077	0.164	-0.399	0.245	-0.468	.640
HY-score	-0.094	0.072	-0.235	0.046	-1.316	.188
creatively active before PD diagnosis	-0.081	0.037	-0.153	-0.009	-2.201	**.028**

Note. Results based on generalized linear model from an exploratory stepwise series (statsmodels python library[27]); 58 individuals reporting creativity fluctuation excluded. Dependent variable: creativity change along three categories (0 = decrease, 1 = no-change, 2 = increase). Independent variables included gender identity, age, education, working situation, living situation, disease duration in years, MoCA, HY-score, and prior creatively active. Gender identity categorized into two groups (women = 1, men = 0); education coded into three ordinal levels (low, medium, high; see Table 1 note for further documentation); working situation coded into two categorical groups (working = 1, not working = 0); living situation coded into two categorical groups (with partner/family = 1, alone/other = 0); MoCA, in line with former studies[35], was divided into two categorical groups with a cut-off value of <= 17 representing cognitive impairments are present (encoded as 1); everything >=18 means no impairment (encoded as 0); HY-scores were coded into five ordinal levels reflecting disease severity. Ethnicity was excluded from the analysis due to very low numbers in one of the categories. See Methods for full description.

Table S5. Sensitivity analysis, group separation dopamine agonists (*n* = 258).

			95% CI			
Fixed effects	Estimate	SE	Lower	Upper	*z*-value	Pr(>|z|)
Intercept 0/1	-1.212	0.449	-2.092	-0.333	-2.701	**.007**
Intercept 1/2	1.058	0.077	0.906	1.209	13.696	**.000**
gender identity (women)	0.545	0.292	-0.028	1.118	1.866	.062
age	-0.023	0.018	-0.059	0.012	-1.279	.201
education	0.237	0.177	-0.110	0.584	1.338	.181
working situation (working)	-0.280	0.381	-1.027	0.466	-0.736	.462
living situation (with relatives)	0.078	0.400	-0.707	0.862	0.194	.846
disease duration	0.042	0.028	-0.013	0.097	1.487	.137
MoCA	-0.242	0.323	-0.875	0.391	-0.749	.454
HY-score	-0.200	0.133	-0.462	0.061	-1.500	.134
creatively active before PD diagnosis	-0.016	0.072	-0.157	0.125	-0.223	.824

Note. Results based on Ordinal regression reporting creativity change along three categories (0 = decrease, 1 = no-change, 2 = increase) as dependent variable. Independent variables included gender identity, age, education, working situation, living situation, disease duration in years, MoCA, HY-score, and prior creatively active. Gender identity categorized into two groups (women = 1, men = 0); education coded into three ordinal levels (low, medium, high; see Table 1 note for further documentation); working situation coded into two categorical groups (working = 1, not working = 0); living situation coded into two categorical groups (with partner/family = 1, alone/other = 0); MoCA, in line with former studies[35], was divided into two categorical groups with a cut-off value of <= 17 representing cognitive impairments are present (encoded as 1); everything >=18 means no impairment (encoded as 0); HY-scores were coded into five ordinal levels reflecting disease severity. Ethnicity was excluded from the analysis due to very low numbers in one of the categories. See Methods for full description.

Table S6. Sensitivity analysis, group separation levodopa only (*n* = 534).

			95% CI			
Fixed effects	Estimate	SE	Lower	Upper	*z*-value	Pr(>|z|)
Intercept 0/1	-0.997	0.305	-1.596	-0.399	-3.266	**.001**
Intercept 1/2	1.259	0.052	1.156	1.361	24.114	**.000**
gender identity (women)	-0.050	0.212	-0.467	0.366	-0.236	.813
age	-0.020	0.014	-0.047	0.008	-1.407	.160
education	0.030	0.120	-0.205	0.266	0.253	.800
working situation (working)	0.068	0.292	-0.503	0.640	0.234	.815
living situation (with relatives)	0.094	0.275	-0.445	0.633	0.341	.733
disease duration	0.042	0.024	-0.005	0.090	1.746	.081
MoCA	-0.001	0.211	-0.415	0.413	-0.003	.997
HY-score	-0.087	0.095	-0.272	0.099	-0.913	.361
creatively active before PD diagnosis	-0.104	0.047	-0.197	-0.011	-2.192	**.028**

Note. Results based on Ordinal regression reporting creativity change along three categories (0 = decrease, 1 = no-change, 2 = increase) as dependent variable. Independent variables included gender identity, age, education, working situation, living situation, disease duration in years, MoCA, HY-score, and prior creatively active. Gender identity categorized into two groups (women = 1, men = 0); education coded into three ordinal levels (low, medium, high; see Table 1 note for further documentation); working situation coded into two categorical groups (working = 1, not working = 0); living situation coded into two categorical groups (with partner/family = 1, alone/other = 0); MoCA, in line with former studies[35], was divided into two categorical groups with a cut-off value of <= 17 representing cognitive impairments are present (encoded as 1); everything >=18 means no impairment (encoded as 0); HY-scores were coded into five ordinal levels reflecting disease severity. Ethnicity was excluded from the analysis due to very low numbers in one of the categories. See Methods for full description.

Table S7. Ordinal regression model predicting increased or decreased creativity changes among people with PD (*n* = 700, excluding participants taking no medication).

Fixed effects	Estimate	SE	95% CI Lower	95% CI Upper	*z*-value	Pr(>|z|)
Intercept 0/1[a]	-0.893	0.252	-1.388	-0.398	-3.538	**<.001**
Intercept 1/2 [a]	1.180	0.043	1.097	1.264	27.698	**<.001**
gender identity (women)[b]	0.159	0.171	-0.175	0.494	0.934	.351
age	-0.023	0.011	-0.045	-0.001	-2.067	**.039**
education[c]	0.098	0.098	-0.095	0.290	0.997	.319
working situation (working)[d]	-0.089	0.229	-0.537	0.360	-0.386	.699
living situation (with relatives)[e]	0.068	0.226	-0.375	0.511	0.302	.763
disease duration	0.040	0.018	0.004	0.075	2.189	**.029**
MoCA[f]	-0.054	0.175	-0.397	0.288	-0.311	.756
disease severity[g]	-0.111	0.077	-0.261	0.039	-1.449	.147
creative lifestyle before PD diagnosis	-0.073	0.039	-0.149	0.004	-1.865	.062
medication (dopamine agonist)[h]	0.707	0.188	0.338	1.075	3.760	**<.001**

Note: Results based on final generalized linear model from an exploratory stepwise series using ordinal regression (statsmodels python library[36]); 58 individuals reporting creativity fluctuation excluded. 35 individuals taking no medication were excluded. [a] Coding of creativity changes into three ordinal levels (0 = decrease, 1 = no-change, 2 = increase); [b] gender identity categorized into two groups (women = 1, men = 0); [c] education coded into three ordinal levels (low, medium, high; see Table 1 note for further documentation); [d] working situation coded into two categorical groups (working = 1, not working = 0); [e] living situation coded into two categorical groups (with partner/family = 1, alone/other = 0); [f] MoCA, in line with former studies[37], was divided into two categorical groups with a cut-off value of <= 17 representing cognitive impairments are present (encoded as 1); everything >=18 means no impairment (encoded as 0); [g] HY-scores were coded into five ordinal levels reflecting disease severity; [h] medication coded into two categorical groups (levodopa only = 0, dopamine agonist = 1). Ethnicity was excluded from the analysis due to very low numbers in one of the categories. See Methods for full description.

Table S8. Ordinal regression analysis examining the interaction significant predictors reported in Table 2 (*N* = 793).

			95% CI			
Fixed effects	Coef	SE	Lower	Upper	*z*-value	Pr(>|z|)
Intercept 0/1	-0.998	0.103	-1.200	-0.796	-9.696	**<.001**
Intercept 1/2	1.199	0.042	1.117	1.281	28.675	**<.001**
age	-0.019	0.012	-0.043	0.004	-1.600	.110
disease duration	0.014	0.024	-0.032	0.060	0.597	.550
creative lifestyle before PD diagnosis	-0.086	0.045	-0.175	0.003	-1.904	.057
medication (dopamine agonist)	0.758	0.192	0.381	1.134	3.945	**<.001**
**Interactions**						
medication*creative lifestyle before PD	0.071	0.094	-0.114	0.255	0.752	.452
medication*disease duration	0.030	0.037	-0.042	0.102	0.813	.416
medication*age	-0.031	0.022	-0.075	0.013	-1.373	.170
creative lifestyle before PD *disease duration	0.016	0.009	-0.001	0.034	1.824	.068
creative lifestyle before PD *age	-0.005	0.005	-0.014	0.005	-0.904	.366
disease duration*age	0.003	0.002	0.000	0.007	1.705	.088

Note: Results based on final generalized linear model from an exploratory stepwise series using ordinal regression (statsmodels python library[35]); 58 individuals reporting creativity fluctuation excluded. Coding of creativity changes into three ordinal levels (0 = decrease, 1 = no-change, 2 = increase); medication coded into two categorical groups (dopamine agonist = 1, levodopa only/no medication = 0). Note, that the levodopa-only and no-medication groups were combined for analysis due to the very low number of individuals in the latter category (*n* = 39) and based on literature suggesting that dopamine agonist use was the main hypothesized factor for creativity change[15].See Methods for full description.

Table S9. Medication use in 2019 nationwide Dutch population (vektis)

Medication Type	N = 24,447	%
No medication	1,631	6.7
Levodopa only	15,269	62.5
Dopamine agonist only	448	1.8
Levodopa and dopamine agonist	7,099	29.0

Table S10. Medication use in 2021 nationwide Dutch population (vektis)

Medication Type	N = 24,447	%
No medication	2,580	10.6
Levodopa only	14,334	58.6
Dopamine agonist only	246	1.0
Levodopa and dopamine agonist	7,287	29.8

Table S11. Prevalence of creativity change along seven creative domains (*n* = 292).

Creative domain [a]	creatively active in domain (excl. never done) (% of total)	no creativity change (% of active)	some creativity change (% of creatively active)	creativity change type			% creativity increase vs. decrease (based on absolute sum increase plus decrease)
				increase (% of active)	decrease (% of active)	fluctuations (% of active)	
fine art/design	203 (69.52%)	28 (13.79%)	175 (86.21%)	59 (29.06%)	82 (40.39%)	34 (16.75%)	41.84%
performing arts/dance	78 (26.71%)	47 (60.26%)	31 (39.74%)	11 (14.1%)	15 (19.23%)	5 (6.41%)	42.31%
literature/writing	138 (47.26%)	39 (28.26%)	99 (71.74%)	32 (23.19%)	48 (34.78%)	19 (13.77%)	40.00%
music/singing/sound	177 (60.62%)	51 (28.81%)	126 (71.19%)	27 (15.25%)	76 (42.94%)	23 (12.99%)	26.21%
sports/movement	185 (70.21%)	54 (29.19%)	151 (81.62%)	43 (23.24%)	88 (47.57%)	20 (10.81%)	32.82%
science/technology	137 (46.92%)	50 (36.5%)	87 (63.5%)	26 (18.98%)	54 (39.42%)	7 (5.11%)	32.50%
everyday creativity	238 (81.51%)	52 (21.85%)	186 (78.15%)	52 (21.85%)	109 (45.8%)	25 (10.5%)	32.30%

Note. Demographics for sub-cohort: M_{age} = 71.01, *SD* = 8.23; age-range = 42 to 91; 52.05 % men, 47.95 % women, ethnicity 99.2% Dutch; M_{MoCA} = 18.40, SD_{MoCA} = 2.79; M_{HY} = 2.66, SD_{HY} = 1.12). Sub-cohort of participants showed *n* = 90 (30.82%) increased creativity as assessed via main survey, *n* = 152 (52.05%) decreased, and *n* = 50 (17.12%) fluctuating. For comparison, individuals who did not answer to these voluntary questions or were not offered to answer showed *n* = 4 (0.80%) increased, *n* = 19 (3.79%) decreased, *n* = 8 (1.60%) fluctuating creativity, and *n* = 470 (93.81%) no reported change.
[a] Creative domain based on the Inventory of Creative Activities and Achievements with sub-items merged into main categories for overall length/time reasons. Participants responded to each domain individually and thus could highlight multiple domains and types of change prevalence/directionality across the domains (see Methods for full description).

Table S12. Sensitivity analysis, group separation PRIME-NL region of patient residence/care (*n* = 300).

			95% CI			
Fixed effects	Estimate	SE	Lower	Upper	*z*-value	Pr(>|z|)
Intercept 0/1	-1.180	0.369	-1.903	-0.457	-3.197	**.001**
Intercept 1/2	1.343	0.067	1.211	1.475	19.904	**<.001**
gender identity (women)	-0.329	0.273	-0.864	0.205	-1.207	.227
age	-0.008	0.018	-0.044	0.028	-0.427	.669
education	-0.175	0.155	-0.480	0.129	-1.128	.259
working situation (working)	0.257	0.409	-0.546	1.059	0.627	.530
living situation (with relatives)	-0.001	0.329	-0.645	0.643	-0.002	.998
disease duration	0.044	0.028	-0.011	0.099	1.575	.115
MoCA	-0.125	0.269	-0.652	0.402	-0.464	.643
HY-score	-0.175	0.119	-0.407	0.058	-1.473	.141
creatively active before PD diagnosis	-0.075	0.062	-0.195	0.046	-1.209	.227
medication	1.060	0.400	0.275	1.844	2.647	**.008**

Note. Results based on Ordinal regression reporting creativity change along three categories (0 = decrease, 1 = no-change, 2 = increase) as dependent variable. Independent variables included gender identity, age, education, working situation, living situation, disease duration in years, MoCA, HY-score, prior creatively active, and medication. Gender identity categorized into two groups (women = 1, men = 0); education coded into three ordinal levels (low, medium, high; see Table 1 note for further documentation); working situation coded into two categorical groups (working = 1, not working = 0); living situation coded into two categorical groups (with partner/family = 1, alone/other = 0); MoCA, in line with former studies[35], was divided into two categorical groups with a cut-off value of <= 17 representing cognitive impairments are present (encoded as 1); everything >=18 means no impairment (encoded as 0); HY-scores were coded into five ordinal levels reflecting disease severity; medication coded into two categorical groups (dopamine agonist = 1, levodopa only/no medication = 0). Note, that the levodopa-only and no-medication groups were combined for analysis due to the very low number of individuals in the latter category (*n* = 39) and based on literature suggesting that dopamine agonist use was the main hypothesized factor for creativity change[15]. For similar model excluding 'no medication' group see Table S7. Ethnicity was excluded from the analysis due to very low numbers in one of the categories. See Methods for full description.

Table S13. Sensitivity analysis, group separation usual care region of patient residence/care (*n* = 435).

			95% CI			
Fixed effects	Estimate	SE	Lower	Upper	*z*-value	Pr(>|z|)
Intercept 0/1	-0.545	0.350	-1.230	0.141	-1.558	**.119**
Intercept 1/2	1.126	0.055	1.019	1.233	20.569	**<.001**
gender identity (women)	0.451	0.217	0.024	0.877	2.072	**.038**
age	-0.031	0.014	-0.058	-0.004	-2.283	**.022**
education	0.324	0.133	0.063	0.585	2.432	**.015**
working situation (working)	-0.201	0.269	-0.729	0.327	-0.746	.456
living situation (with relatives)	0.118	0.311	-0.492	0.728	0.378	.705
disease duration	0.036	0.024	-0.011	0.083	1.491	.136
MoCA	-0.009	0.227	-0.453	0.435	-0.039	.969
HY-score	-0.118	0.096	-0.306	0.070	-1.228	.219
creatively active before PD diagnosis	-0.115	0.049	-0.211	-0.019	-2.358	**.018**
medication	0.671	0.219	0.241	1.101	3.059	**.002**

Note. Results based on Ordinal regression reporting creativity change along three categories (0 = decrease, 1 = no-change, 2 = increase) as dependent variable. Independent variables included gender identity, age, education, working situation, living situation, disease duration in years, MoCA, HY-score, prior creatively active, and medication. Gender identity categorized into two groups (women = 1, men = 0); education coded into three ordinal levels (low, medium, high; see Table 1 note for further documentation); working situation coded into two categorical groups (working = 1, not working = 0); living situation coded into two categorical groups (with partner/family = 1, alone/other = 0); MoCA, in line with former studies[35], was divided into two categorical groups with a cut-off value of <= 17 representing cognitive impairments are present (encoded as 1); everything >=18 means no impairment (encoded as 0); HY-scores were coded into five ordinal levels reflecting disease severity; medication coded into two categorical groups (dopamine agonist = 1, levodopa only/no medication = 0). Note, that the levodopa-only and no-medication groups were combined for analysis due to the very low number of individuals in the latter category (*n* = 39) and based on literature suggesting that dopamine agonist use was the main hypothesized factor for creativity change[15]. For similar model excluding 'no medication' group see Table S7. Ethnicity was excluded from the analysis due to very low numbers in one of the categories. See Methods for full description.

Figures

Fig. S1. Correlation heatmap

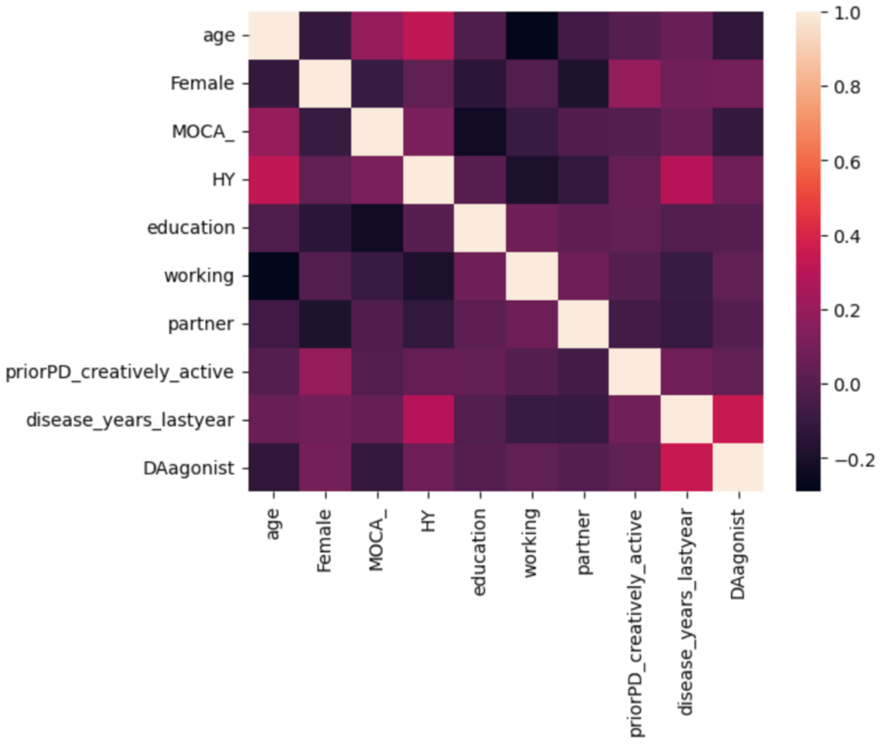

Fig S2. Distribution of self-reported creativity change by medication type levodopa only/no-medication and dopamine agonists

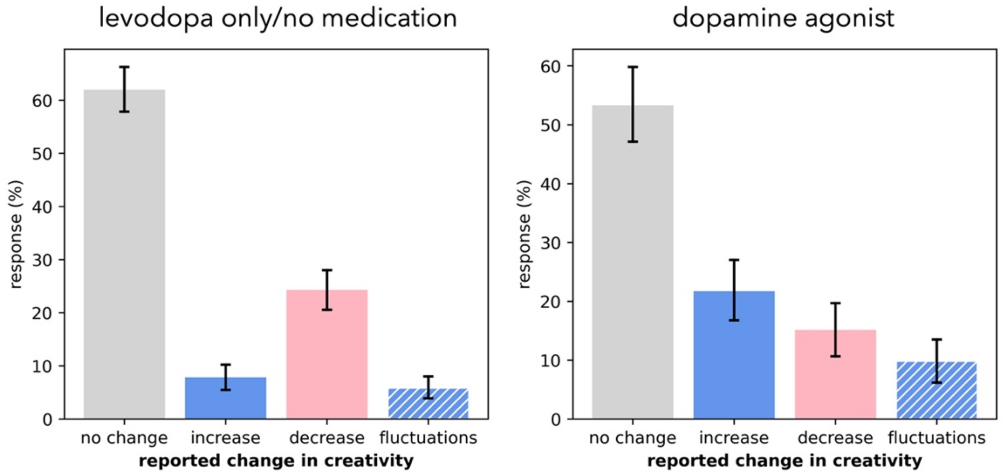

Fig S3. Distribution of self-reported creativity change by age, prior creative lifestyle, and disease duration)

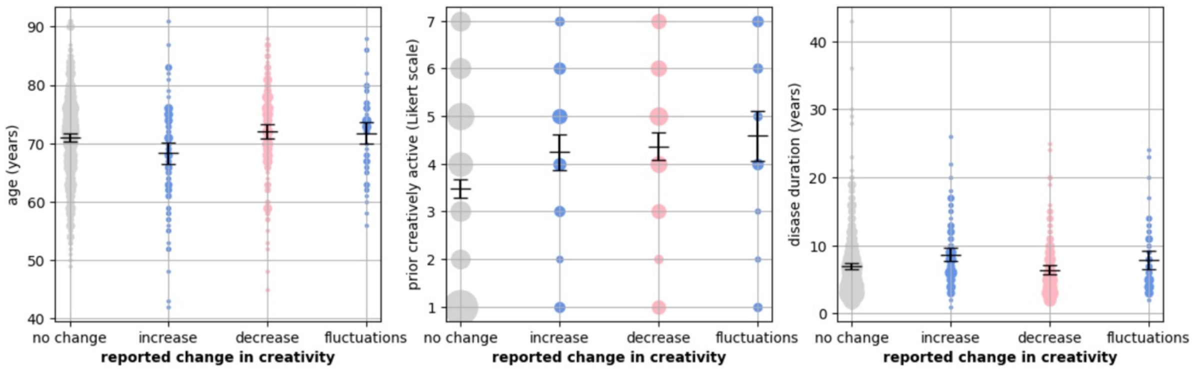

Supplement: Supplementary file 1 — Supplementary Materials [file 41531_2025_924_MOESM1_ESM.pdf]
